# Whole-Genome Sequence of *Bifidobacterium longum* Strain Indica, Isolated from the Gut of a Healthy Indian Adult

**DOI:** 10.1128/genomeA.01017-17

**Published:** 2017-10-12

**Authors:** Satyabrata Bag, Tarini Shankar Ghosh, Bhabatosh Das

**Affiliations:** Molecular Genetics Laboratory, Centre for Human Microbial Ecology, Translational Health Science and Technology Institute, NCR Biotech Science Cluster, Faridabad, India

## Abstract

*Bifidobacterium longum*, a Gram-positive rod-shaped anaerobic bacterium, inhabits the human gastrointestinal tract and contributes significantly to oligosaccharide production, amino acid metabolism, and protection against intestinal inflammation. Here, we report the whole-genome sequence of *B. longum*, which was isolated from the gastrointestinal tract of a healthy Indian adult.

## GENOME ANNOUNCEMENT

Different species of bifidobacteria play important roles in the homeostasis of the gut microbiome. Recent metagenomic studies of the human gut microbiota revealed that, while *B. longum*, *B. bifidum*, and *B. breve* are mostly dominant in infants, *B. adolescentis* and *B. catenulatum* are more prevalent in adults (1). Depletion of *B. longum* at an early age leads to gut microbial dysbiosis and is linked to health disorders such as severe acute malnutrition (SAM) (2, 3). To assess the efficacy of *B. longum* as a universal probiotic and its contribution in host physiology, it is important to explore the genome sequences of *B. longum* isolates from different parts of the world.

*B. longum* strain Indica was isolated from the fecal samples of a healthy Indian adult. The fresh fecal sample was immediately resuspended in prereduced phosphate-buffered saline, serially diluted, and then plated onto a tryptic soy agar (Difco) plate supplemented with sterile defibrinated sheep blood 5% (vol/vol). The plates were incubated for 24 h at 37°C in an anaerobic workstation (Whitley DG250) filled with a mixture of anaerobic gases (80% N_2_, 10% CO_2_, 10% H_2_).

Genomic DNA of *B. longum* Indica was extracted by using THSTI methods (4). Whole-genome shotgun libraries were constructed by using approximately 1 µg of genomic DNA. To generate DNA fragments of 1.5-kb average length, the genomic DNA was nebulized at 15 lb/in^2^ for 60 s. DNA fragments of the desired length were then end polished by using T4 DNA ligase, polynucleotide kinase, and *Taq* DNA polymerase (Roche, USA) and ligated with a sequencing adaptor (Roche, USA). Genomic DNA libraries were then purified using AMPure XP beads (Beckman Coulter, Inc., USA) and quality checked using the high-sensitivity DNA chip compatible with the 2100 Bioanalyzer (Agilent, USA). The total amounts of double-stranded DNA in the libraries were quantified using PicoGreen dye with a Qubit fluorometer (Invitrogen, USA). Approximately 1.0 × 10^8^ DNA molecules per sample were amplified by emulsion PCR using Mastercycler proS PCR systems (Eppendorf, Germany). The amplified products were purified using Biomek 3000 (Beckman Coulter, Inc., USA). Sequencing was performed in picotiter plates using GS-FLX+ genome sequencers (Roche, USA).

Whole-genome sequencing of *B. longum* produced a total of 591,026 reads (total volume of 427.43 Mb) with a 941-bp modal read length and 60% GC content. Assembly of the reads using the GS *de novo* version 2.3 assembler produced 28 scaffolds with a highest read length of 495,967 bp. Annotation of the scaffolds was performed using the NCBI Prokaryotic Genome Annotation Pipeline (released 2013). Analysis of 28 scaffolds identified 2,123 coding sequences, including (i) 63 RNA-encoding genes, (ii) 190 genes linked with carbohydrate metabolism, (iii) 29 resistance genes encoding resistance to antibiotics and toxic compounds, and (iv) 13 genes associated with mobile genetic elements, such as plasmids, transposable elements, and prophages. The genome sequence of strain Indica will contribute to a better understanding of the biology of *B. longum*, including its protective role in inflammatory bowel disease and SAM, as well as the molecular basis of its dominance in the gut of infants.

### Accession number(s).

This whole-genome shotgun project was deposited at DDBJ/ENA/GenBank under the accession number NQMV00000000. The version described here is the first version, NQMV01000000.
